# Web GIS in practice VII: stereoscopic 3-D solutions for online maps and virtual globes

**DOI:** 10.1186/1476-072X-8-59

**Published:** 2009-10-22

**Authors:** Maged N Kamel Boulos, Larry R Robinson

**Affiliations:** 1 Faculty of Health, University of Plymouth, Drake Circus, Plymouth, Devon PL4 8AA, UK; 2 Geospatial Sciences and Technologies Branch, USGS-BRD-Upper Midwest Environmental Sciences Center, 2630 Fanta Reed Road, La Crosse, Wisconsin 54603, USA

## Abstract

Because our pupils are about 6.5 cm apart, each eye views a scene from a different angle and sends a unique image to the visual cortex, which then merges the images from both eyes into a single picture. The slight difference between the right and left images allows the brain to properly perceive the 'third dimension' or depth in a scene (stereopsis). However, when a person views a conventional 2-D (two-dimensional) image representation of a 3-D (three-dimensional) scene on a conventional computer screen, each eye receives essentially the same information. Depth in such cases can only be approximately inferred from visual clues in the image, such as perspective, as only one image is offered to both eyes. The goal of stereoscopic 3-D displays is to project a slightly different image into each eye to achieve a much truer and realistic perception of depth, of different scene planes, and of object relief. This paper presents a brief review of a number of stereoscopic 3-D hardware and software solutions for creating and displaying online maps and virtual globes (such as Google Earth) in "true 3D", with costs ranging from almost free to multi-thousand pounds sterling. A practical account is also given of the experience of the USGS BRD UMESC (United States Geological Survey's Biological Resources Division, Upper Midwest Environmental Sciences Center) in setting up a low-cost, full-colour stereoscopic 3-D system.

## Background

Stereoscopic 3-D (three-dimensional) visualization is often confused with the conventional display of 3-D objects and scenes on a computer screen or other media. In stereoscopic 3-D rendering, there is a much truer perception of depth (the 'third dimension'), of different scene planes, and of object relief, as each eye is actually presented with a slightly different image of the same scene, while in conventional (pseudo or "flat") 3-D presentations, depth can only be approximately simulated at best, as only one image is offered to both eyes.

Most humans have the capability to perceive and measure depth with two eyes using binocular (stereo) vision. Stereopsis is the physiological process in visual perception in which the human brain synthesizes and fuses the two slightly different projections of the world onto the retinae of the two eyes to create this sensation of depth [1]. When viewing imagery and vector data using stereoscopic 3-D vision the interrelationships between features and the real world become clearer, photo interpretation becomes more accurate and complete, and spatial accuracy is increased [2].

The hardware and software technologies for stereoscopic 3-D visualization on computer screens have been significantly perfected and made more affordable over the past decade, as we will briefly see in this paper. There has also been a parallel growing (and renewed) interest in recent years in stereoscopic "true 3-D" computer geodata presentations, as evidenced, for example, by the launch in August 2009 of a new conference series dedicated to the subject [3].

## Technologies for stereoscopic 3-D visualization

### Passive 3-D glasses

**Figure 1:**
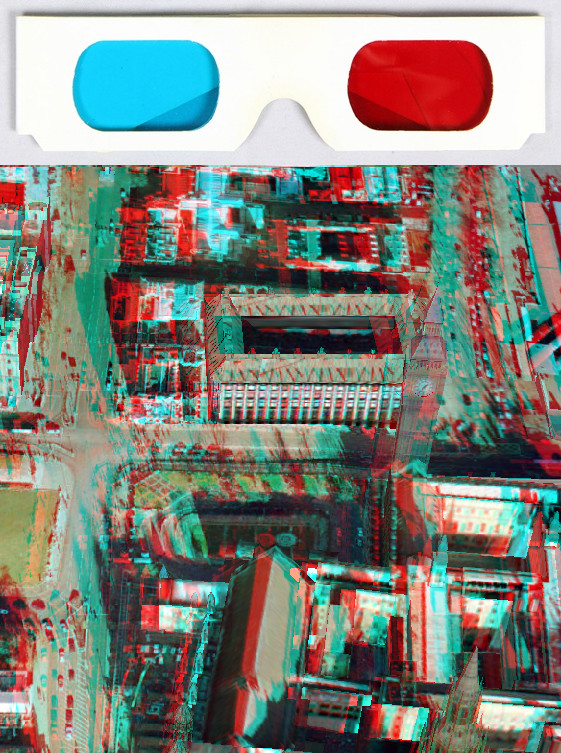
**A pair of red/cyan 3-D glasses made of cardboard and a red/cyan anaglyph image (half colour) suitable for viewing by those glasses**.

**Figure 2:**
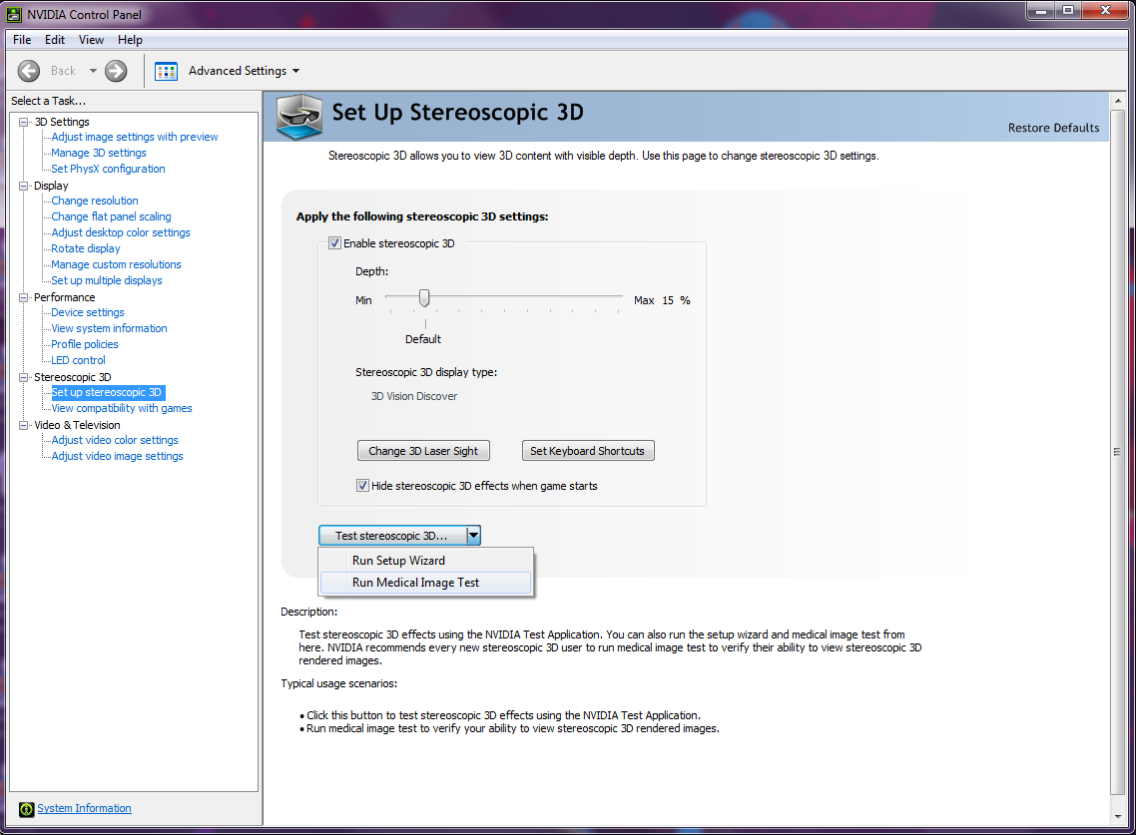
**NVIDIA Stereoscopic 3D options (located in NVIDIA graphics card's Control Panel) include an on-screen 'Medical Test'**.

This method uses simple and low-cost two-colour glasses (where each lens is of a chromatically opposite colour, usually red for left eye and cyan for right eye) to restrict the light that reaches each eye. Stereoscopic 3-D images compatible with this technique are known as anaglyph images. They are made up of two "colour-coded", slightly different perspectives of the same scene (e.g., a red/cyan stereoscopic image pair), one for each eye, superimposed, but offset with respect to one another to produce a depth effect when viewed with a matching pair of glasses (Figure 1). Anaglyph images do not require any special monitor to properly display them, adding to the technique's appeal as a low-cost stereoscopic 3-D solution. Examples of graphics-driver-level implementations of this technique include NVIDIA 3D Vision Discover (a free, low-end version of NVIDIA 3D Vision running in 'half colour anaglyph mode with colour correction shader'--Figure 2) [4] for NVIDIA GPUs (Graphics Processing Units) and iZ3D driver (set to the free '3D anaglyph output mode for DirectX/Direct3D') [5] for ATI and NVIDIA GPUs.

The main problems with anaglyphs are ghosting, retinal rivalry, and limited colour fidelity. Solutions exist for reducing ghosting and retinal rivalry (see examples at [6,7]), but colours can never be reproduced correctly in anaglyph images [8]; for example, red can only be rendered as dark purple-brown, at best, in a red/cyan anaglyph.

Another form of passive 3-D glasses viewing utilises special 3-D polarized LCD (Liquid Crystal Display) monitors and passive (non-coloured) polarized glasses, e.g., Zalman 3D Monitors (using interlaced polarization technology) [9], the iZ3D Monitor (employing dual stacked panels technology for full 3D resolution, with first panel controlling the pixel intensity and the second controlling polarization orientation) [10,11], and Planar's SD monitors (StereoMirror™ technology) [12]. Polarized light travels in waves rolling in the same orientation. Different orientations can be used to encode the left and right channels of a stereoscopic image pair. The polarized glasses worn by the user then act as filters for channel separation; each lens only allows polarized light of matching orientation to pass through. Colour fidelity is preserved with this method. This technology costs about £200-250 (GBP) per 22" 3-D monitor with matching polarized glasses (consumer prices as of October 2009), except for Planar's StereoMirror monitors, which cost significantly much more (£2500 for the 22" SD2220W version - consumer price in GBP as of October 2009).

### Active 3-D (shutter) glasses

This technology uses a pair of battery powered, wireless LCD shutter glasses in conjunction with a special, 3-D-Vision-Ready, high-refresh-rate (120 Hz) display screen [13]. Each lens in the active shutter glasses becomes dark when voltage is applied, but otherwise is transparent. The glasses have a built-in infrared (IR) receiver and are controlled wirelessly by an IR emitter connected to the computer via USB (Universal Serial Bus). They alternately darken over one eye, and then the other, in synchronization with the refresh rate of the monitor, while the display alternately displays different perspectives for each eye, using a technique called alternate-frame sequencing. NVIDIA 3D Vision [14] (not to be confused with NVIDIA 3D Vision Discover (anaglyph) [4]; see product comparison table at [15]) employs this method for computers with compatible NVIDIA GPUs. The technology offers full colour fidelity. NVIDIA 3D Vision Bundle (one pair of glasses, IR emitter, and a 3-D-Vision-Ready 22" 120 Hz LCD monitor) costs about £350-380 (GBP consumer prices as of October 2009).

### Naked-eye (no glasses, autostereoscopic) 3-D displays

Opel and Bergmann [16] provide an overview of recent developments in autostereoscopic display technologies. A number of companies, including SeeReal Technologies [17], Dimension Technologies Inc. [18], Alioscopy [19], and Philips 3D Solutions [20], have introduced their own autostereoscopic 3-D monitors that do not require users to wear special 3-D glasses to view 3-D content.

The ones produced by Philips use a slanted multi-view lenticular lens technology (*cf*. 3-D lenticular postcards and posters) to ensure full picture brightness and contrast, and true colour representation, and also allow multiple users to view 3-D content at the same time within a large comfort zone. A key component in this technology is a sheet of lenticules (transparent cylindrical lenses) fixed on a liquid crystal display. (Other technologies, such as the parallax barrier, block light and therefore greatly reduce brightness.) Format wise, Philips chose a '2D-plus-Depth' approach to store a greyscale 'depth map' representation of an image side-by-side with the 'real' colour image. Depth values in shades of grey are used to render a 3-D representation on the 3-D display; the lighter an area is in the depth map, the closer it will appear to the viewer. (Actually, the information in the 'depth map' is used to transform the original colour frame into nine separate and slightly-differing interlaced images or perspectives, and of these, only two, i.e., a stereo image pair, e.g., the first and third, or third and fifth images, will reach the viewer through the lenticular lens at any one time depending on the viewing angle.) Philips autostereoscopic displays also support a unique Declipse image format that enables a true 'look-around' effect, allowing viewers to perceive slightly different sections of the background behind a foreground object when they change their viewpoint. Philips technology supports both DirectX and OpenGL 3-D applications under Microsoft Windows [20-22].

The 42" version of Philips autostereoscopic LCD monitor costs about £6000-9000 depending on the seller (GBP consumer prices as of October 2009). Other technology limitations besides the high cost are that the 3-D effect works most effectively only up to a distance of about 3.5 meters (with a 42" display) and garbled images might be experienced when the screen is viewed from certain angles [22]. Sadly, Philips decided to discontinue its autostereoscopic 3-D display business in March 2009, stating as reasons the global recession and a slower adoption of the technology than the company had previously hoped for [23].

Glasses-free 3-D viewing is now also possible on mobile devices such as the Apple iPod Touch and iPhone using interlacing software and a removable lenticular lens that allows full use of the Apple touch screen [24]. Other mobile device examples with autostereoscopic 3-D screens include the recently launched Fujifilm FinePix REAL 3D W1 camera, which features a built-in 2.8" autostereoscopic LCD monitor, as well as an optional external 8" autostereoscopic digital viewer, the FinePix REAL 3D V1 (parallax barrier technology) [25]. Furthermore, 3 M came up with its own glasses-free solution for mobile LCD displays up to 9" wide, using directional backlight technology in the form of a special optical film integrated into the display's backlight module to project left and right images sequentially at 120 Hz refresh rate without sacrificing brightness or resolution [26].

A comparison of stereoscopic 3-D technologies, including other technologies not covered in this brief précis such as head mounted displays (*cf*. the View-Master), is available from Planar Systems, Inc. at [27]. Although written by a commercial provider of 3-D monitors (Planar), the information is still generally useful, despite being incomplete in some areas and having some commercial bias in it, e.g., there is no proper mention of the cost of different technologies.

Common to all viewing methods are some eye conditions that can prevent a person from properly seeing stereoscopic 3-D content, e.g., one-eyed persons, people with amblyopia and those with strabismus. In fact, the NVIDIA 3D Vision Setup Wizard (Figure 2) includes a Medical Test step to verify user's ability to view stereoscopic 3-D content.

## Software for stereoscopic 3-D online maps and virtual globes

### StereoPhoto Maker

Written by Masuji Suto and David Sykes, StereoPhoto Maker (SPM) is a free software programme for Microsoft Windows that functions both as a versatile stereo image editor and stereo image viewer [28]. SPM can open and save stereo image pairs in JPEG Stereo (.JPS) format, a JPEG (Joint Photographic Experts Group)-based format for storing two static images side-by-side in a single file, with commonly the left-eye view on the right and the right-eye view on the left. SPM can also open and save images in MPO (multiple picture format), a format used by Fujifilm FinePix REAL 3D W1 camera [25]. It can convert .MPO images to .JPS or save them as separate left and right image files in a number of formats such as JPEG.

SPM can generate stereo views in a wide range of stereo formats such as various types of colour anaglyphs [7] (without the need for any additional 3-D driver) from any suitable stereo image pair (e.g., from a .JPS or an .MPO file or separate left and right JPEG files). The generated stereo views can in turn be saved in a number of formats from within SPM. 'No compression ghosting' can be used to disable chroma subsampling when saving anaglyph JPEG images to eliminate the compression-ghosting associated with standard JPEG compression. SPM can make Flash stereo slideshows that offer viewers multiple stereo formats to choose from. It can also create slideshows that run using a free StereoPhotoViewer Java applet embedded in a Web page and again featuring a wide range of viewer-selectable stereo formats [29].

**Figure 3:**
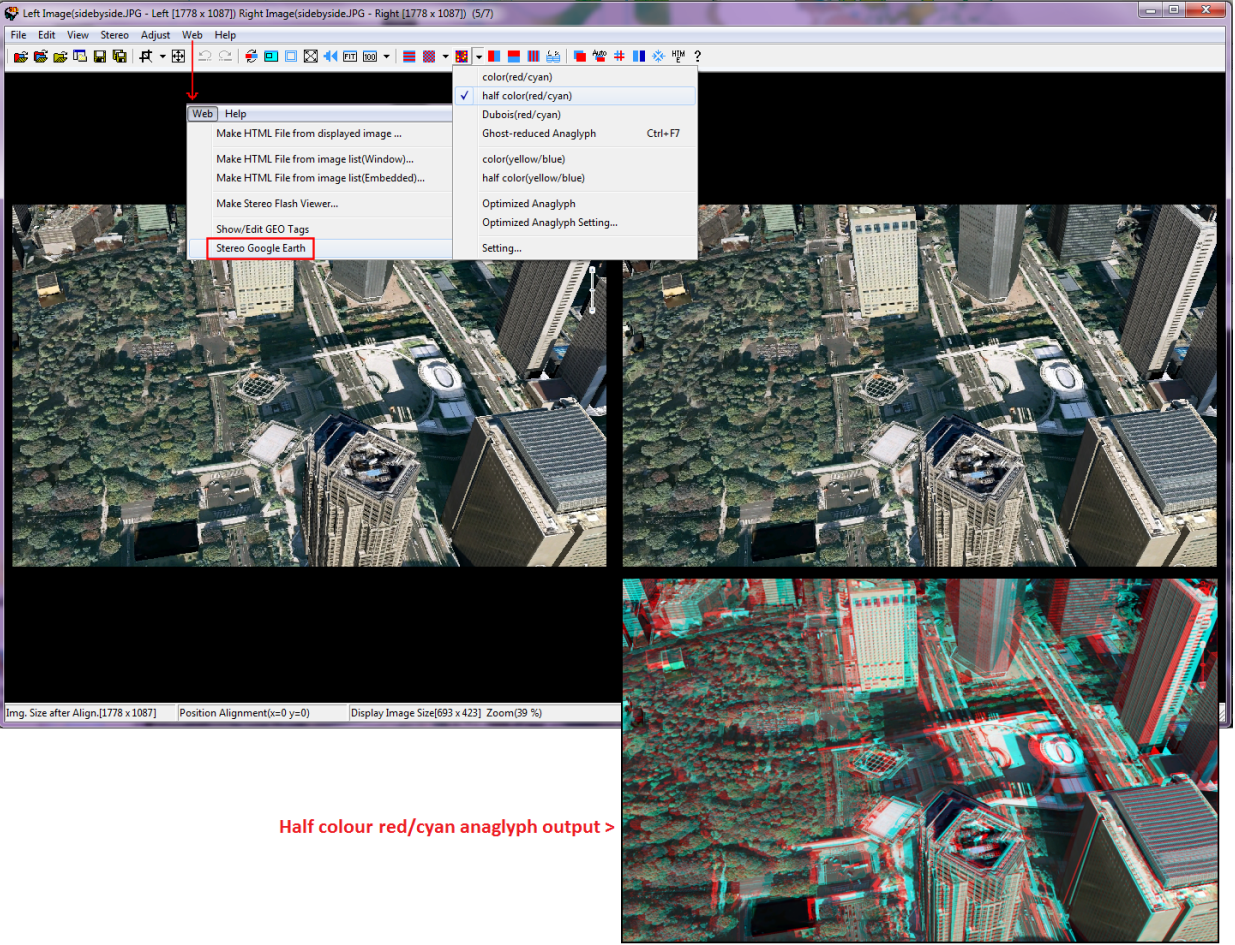
**Screenshot of StereoPhoto Maker**. Screenshot of StereoPhoto Maker [28] showing a side-by-side stereo image pair of a Google Earth location (Tokyo, Japan) that was captured using StereoPhoto Maker's built-in 'Stereo Google Earth' function. Inset (lower right): shows the corresponding half colour (red/cyan) anaglyph stereo image produced by StereoPhoto Maker.

SPM has two geographical functions of interest. 'Show/Edit GEO tags' allows users to write, delete or change a geographical information tag (metadata) associated with an image [30]. The stored geographical location can be shown in a built-in Google Maps/Google Earth window. The second function, 'Stereo Google Earth' (Figure 3), can be used to capture a stereo image pair of a geographical location in Google Earth (using a built-in Google Earth window) [31]. Users can control the stereo base of the captured pair (left-right 'virtual camera' separation). If the stereo base is too big, the stereoscopic 3-D effect will be lost because of the large differences between the resulting left and right images, leaving the brain unable to properly register and fuse them (this is similar to eye conditions where both eyes do not work together correctly).

Harbour [32] describes a simple method whereby images are exported from ArcScene and converted into stereo 3-D anaglyphs using StereoPhoto Maker. (ESRI ArcGIS 3D Analyst extension is a tool for visualizing and analysing surface data. At the core of it, ArcScene provides the interface for viewing layers of 3-D data and creating and analysing surfaces.)

### On-the-fly anaglyph rendering of virtual globes

**Figure 4:**
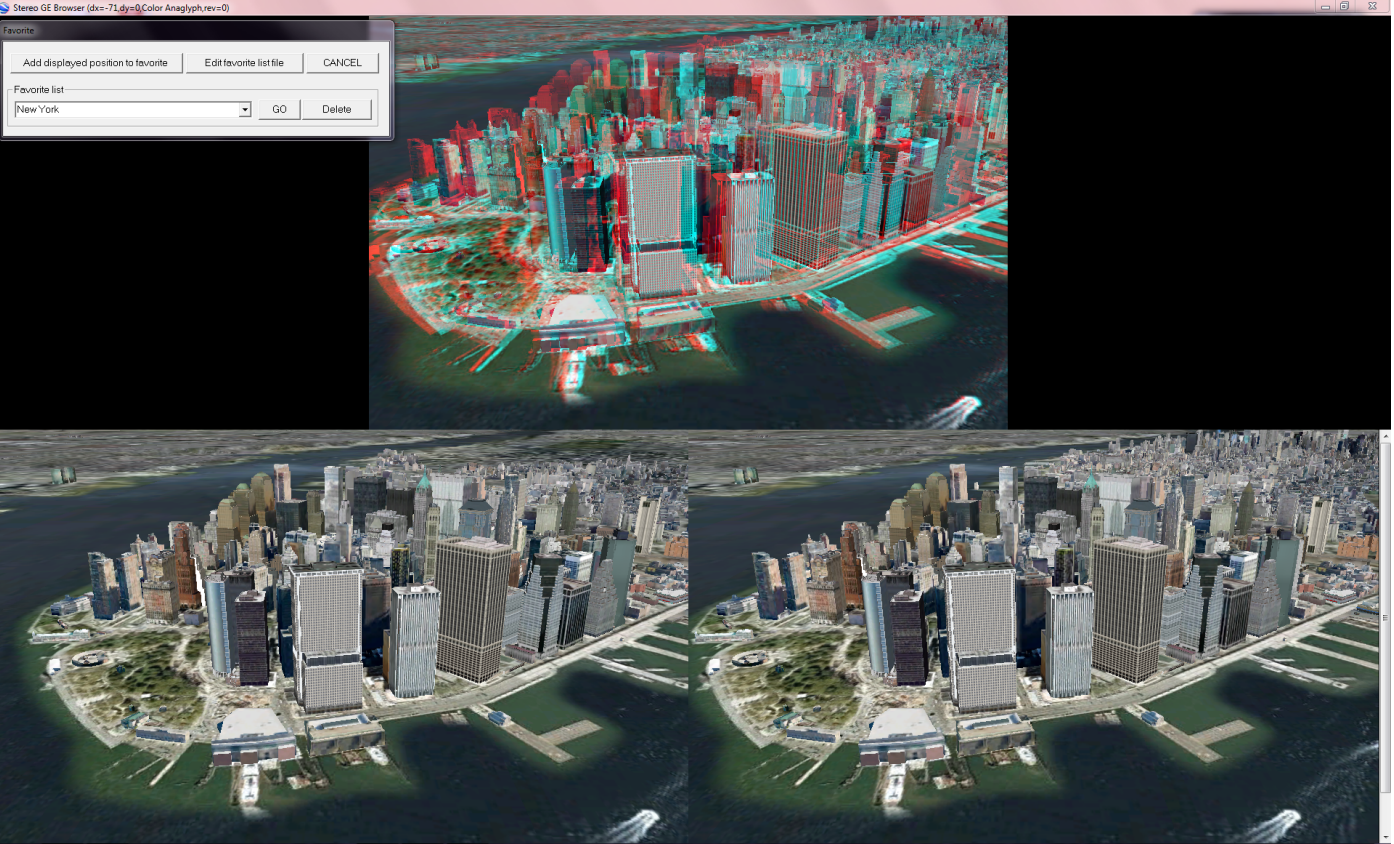
**Screenshot of Stereo GE Browser **[33].

Developed by the same creators of StereoPhoto Maker, Stereo GE Browser [33] is a freeware stereoscopic browser of Google Earth that uses the free Google Earth Browser Plugin from Google [34]. Stereo GE Browser features three synchronized instances of Google Earth (Figure 4); one instance shows the stereo view and two other instances below it display the corresponding left and right views. Users are able to navigate Google Earth as usual by manipulating any of the lower two instances (the two other views will automatically refresh their content). Users can select the stereo viewing method, e.g., half-colour anaglyph, and adjust the stereo base. Other options for real-time stereoscopic viewing in Google Earth include TriDef's Visualizer for Google Earth (part of Dynamic Digital Depth--DDD's TriDef 3D Experience package), which supports anaglyph and other stereo output options, but unlike Stereo GE Browser is not free (a single TriDef 3-D Experience package license costs US $49.99 - Web price as of October 2009) [35].

**Figure 5:**
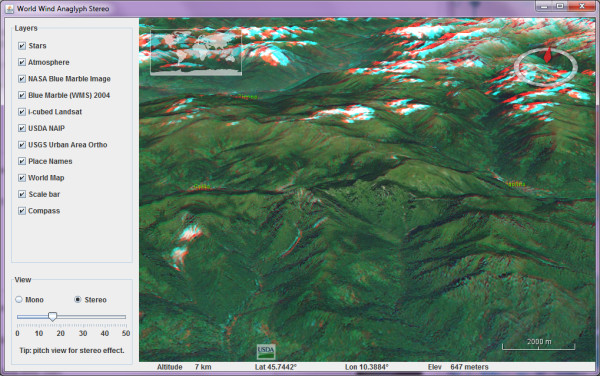
**Screenshot of NASA World Wind Anaglyph Stereo Java application **[37].

**Figure 6:**
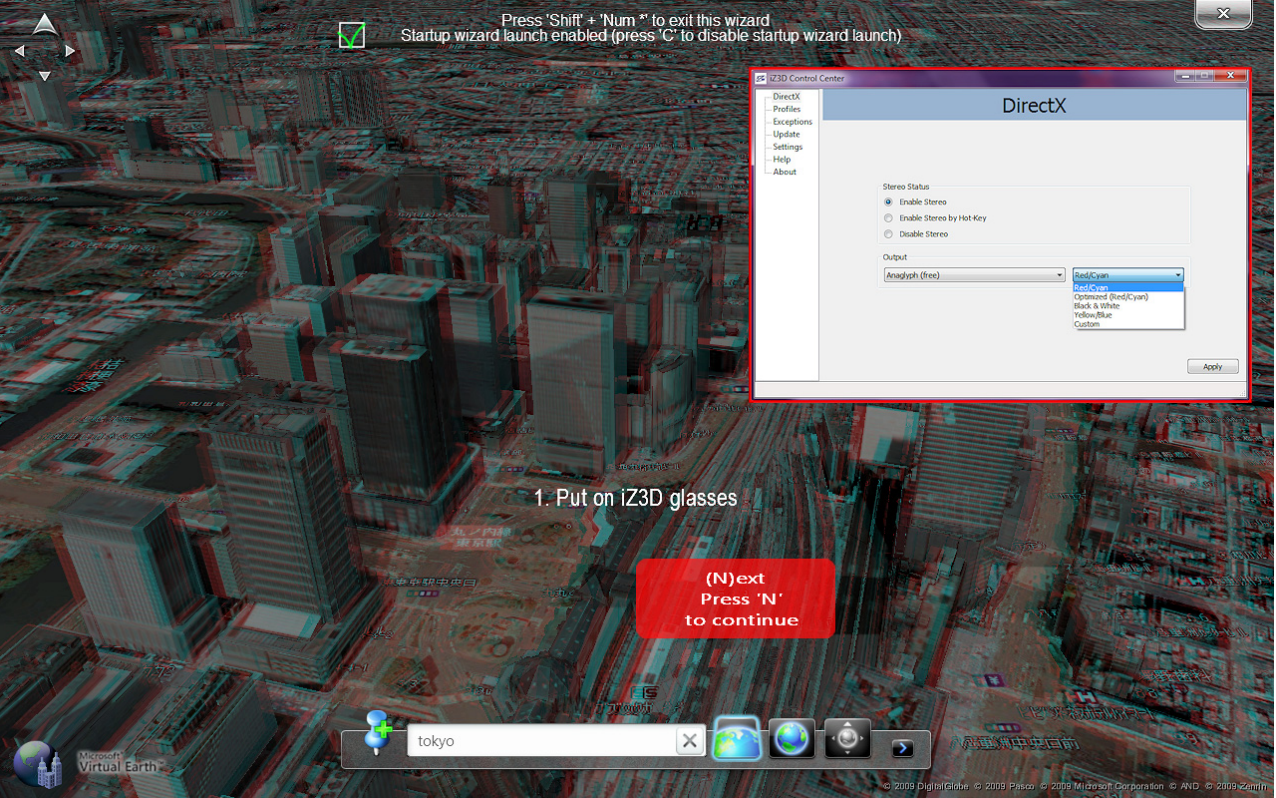
**Stereoscopic 3-D virtual Earth with multitouch navigation**. Screenshot of the multitouch-enabled Microsoft Surface Globe, part of Microsoft Touch Pack for Windows 7 [39], running on a Hewlett-Packard TouchSmart tx2 Notebook PC. The tx2 notebook features a 12.1" multitouch display that allows the use of two or more fingers to navigate multitouch-enabled applications. Microsoft Surface Globe is shown rendered in anaglyph (red/cyan) stereo, with the iZ3D driver [5] wizard superimposed on it. The wizard helps users calibrate separation-convergence to get an optimal stereo image. Inset (upper right): shows the 'iZ3D Control Center (DirectX)' where users can select from a number of anaglyph output options supported by the driver.

Google Earth is not the only virtual globe application to receive dynamic stereoscopic viewing options. NASA World Wind [36] has a free 'Anaglyph Stereo' Java application (Figure 5) [37]. There is also a novel stereoscopic version of NASA World Wind that supports multitouch navigation (developed by Johannes Schöning and Florian Daiber at the Institute for Geoinformatics, University of Münster, Germany) [38]. Kamel Boulos was able to achieve the same on a Hewlett-Packard TouchSmart tx2 Notebook PC running Microsoft Windows 7 operating system with multitouch functionality. This was done in Microsoft Surface Globe, part of Microsoft Touch Pack for Windows 7 [39]. Microsoft Surface Globe is a multitouch-enabled virtual globe (based on Microsoft Virtual Earth/Bing Maps 3D) that runs as a fullscreen DirectX application, and thus can be viewed in stereoscopic 3-D using the free '3D anaglyph output mode for DirectX/Direct3D' of the iZ3D driver [5] (Figure 6) or the free NVIDIA 3D Vision Discover driver (anaglyph) [4] for computers with a compatible NVIDIA GPU.

### Other geospatial/photogrammetry tools of interest

These include:

- StereoGIS [40] from SimWright, Inc., an application that enables the user to analyse imagery of a given area in a 3-D stereo format and easily extract, edit, and/or create 2-D or 3-D vector data products, and high quality digital elevation models from the imagery;

- PurVIEW for ArcGIS [41] from I.S.M. International Systemap Corp., an extension that converts Arc- desktops into precise stereo-viewing windows for geo-referenced aerial or space-borne imagery;

- ERDAS LPS, an integrated collection of software tools for transforming raw imagery into reliable data layers required for all digital mapping, GIS analysis and 3-D visualization. LPS supports a number of add-on modules, including LPS Stereo [42], an add-on for extracting geospatial content using stereoscopic image viewing. Other relevant offerings from ERDAS include Stereo Analyst ERDAS IMAGINE and for ArcGIS [43,44], a tool for collecting 3-D features using stereo visualization; and

- Summit Evolution [45] from DAT/EM Systems International, a stereo-enabled system for performing 3-D feature collection directly into AutoCAD or ArcGIS.

The next section provides a practical account of the experience of the USGS BRD UMESC (United States Geological Survey's Biological Resources Division, Upper Midwest Environmental Sciences Center [46]) in setting up a low-cost, full-colour stereoscopic 3-D system.

## The USGS BRD UMESC experience

**Figure 7:**
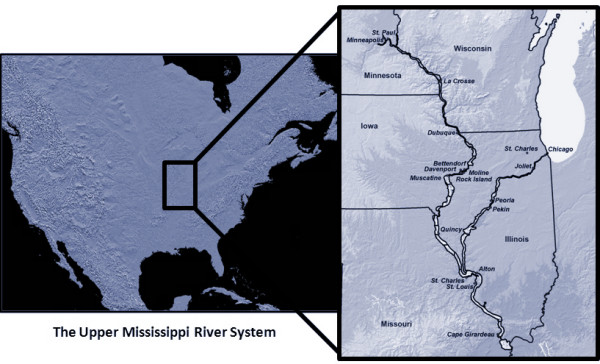
**The Upper Mississippi River System**.

The USGS BRD UMESC [46] has been mapping land use/land cover (LU/LC) for two decades. The goals of these mapping efforts have been to develop a baseline database of habitat conditions on the Upper Mississippi River System (UMRS) and to continue habitat monitoring so that it becomes possible to analyse and assess changing conditions using these spatial data products and status and trends reporting. The UMESC is charged to monitor and inventory the UMRS by virtue of the 1986 Water Resources Development Act [47]. This bill authorized the Environmental Management Program (EMP) [48], and is administered by the US Army Corps of Engineers (USACOE). The UMRS covers nearly 1.2 million hectares and includes the navigable portions of the Mississippi River from Minneapolis, Minnesota to the confluence of the Ohio River, its major tributaries, and the entire reach of the Illinois River (Figure 7).

For the past two decades, this mission to inventory fish and wildlife habitat has used 9" × 9" (23 cm × 23 cm) analogue aerial photography as its primary source of LU/LC data. The original film was separated into individual frames, and a protective acetate overlay was interpreted on a zoom stereoscope with a fine-tipped technical pen. These overlays were scanned into a computer, georeferenced to the Earth, converted to a vector format (with each unit of vegetation given a unique descriptor), and mosaicked into a single database for use in a GIS (Geographic Information System). It can be an expensive and laborious process, but is also established and repeatable. USGS BRD UMESC used essentially the same process to map the floodplain in 2000 but increased the photo scale from 1:15,840 to 1:24,000 and simplified the vegetation classification system to more generalized classes. The next systemic LU/LC inventory is scheduled for 2010 but will use a completely different process.

USGS BRD UMESC is always looking for ways to make this process more efficient and less expensive, while maintaining a high level of cartographic accuracy. With aerial film, and the chemicals necessary to process that film, becoming harder to procure, the USGS BRD UMESC team has considered a switch from a largely analogue workflow to one that would be almost completely digital. This would entail acquiring future aerial photography in a digital format and scanning existing analogue imagery into a digital format. Aerial triangulation software would be used to establish each photo's relationship to other photos in the project and to the Earth's surface. This information would be used to develop stereo models, which can then be used to display stereo pairs of aerial photos in 3-D on compatible computer monitors.

USGS BRD UMESC's first purchase was a 20" (51 cm) stereo mirror display that used two LCD screens, a beam-splitter mirror, image flipping hardware, and passive polarized glasses to view imagery in 3-D (similar to [12]). Though a 4:3 20" display is now considered smaller than ideal, and was quite expensive at the time of purchase (over US $5000), it generated a clear, sharp, high-definition image. Since six stereo monitors were needed, the cost of the stereo mirror display technology prohibited the team from moving forward with this transition.

Early in 2009 news came about a new stereo monitor capable of 120 Hz refresh rates that used active shutter glasses to send separate images to each eye 60 times a second, a rate necessary to avoid any perception of flickering [13,14]. This monitor was designed for 3-D gaming but a driver supporting quad-buffered stereo was promised for the spring of 2009. This was a 22" (56 cm) widescreen monitor with a retail price of US $400, less than a tenth the price of the smaller and bulkier stereo mirror-based 3-D monitor. The active shutter glasses and IR emitter cost an additional US $200, but the combination was still low enough to pair with six NVIDIA Quadro FX 3700-based workstations [49] if the technology worked as promised.

In February, 2009, the USGS BRD UMESC team purchased an NVIDIA 3D Vision monitor and GeForce GTX 260 video card [50] for testing and received special permission to run a dual boot operating system configuration (64-bit versions of Microsoft Windows XP Professional and Windows Vista) since XP was not supported at that time. The US government has strict information technology (IT) requirements, and the team was informed that an XP driver would be necessary if this monitor was to be a viable enterprise option, but having a Vista-capable workstation would allow a 'sneak preview' of viewing stereo pairs of aerial photos on-screen.

**Figure 8:**
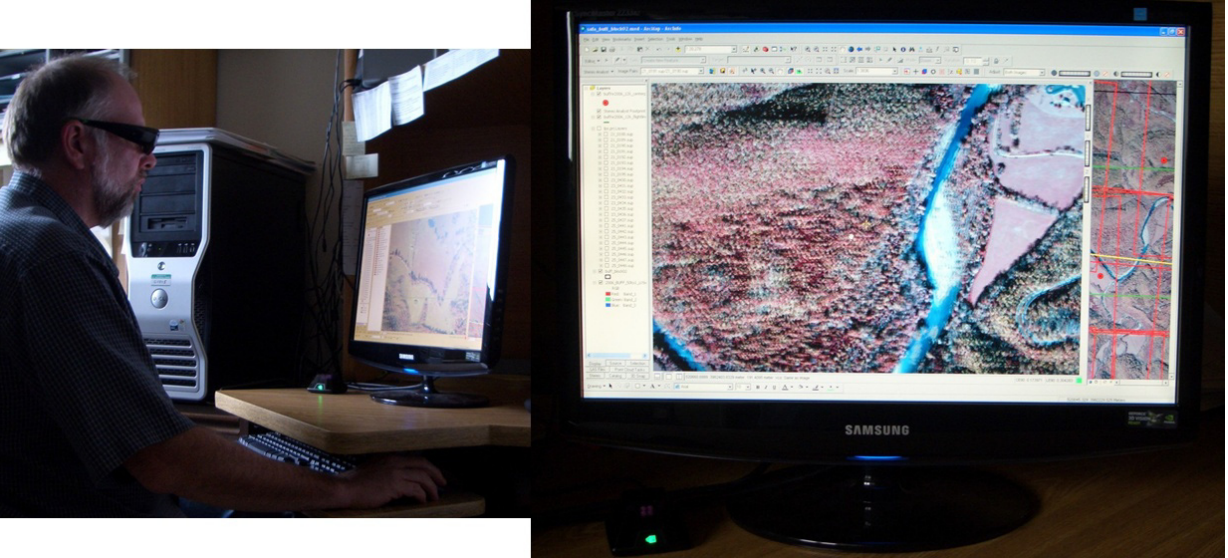
**Stereoscopic 3-D system using NVIDIA 3D Vision Bundle **[14]** and Stereo Analyst for ArcGIS **[44]**at USGS BRD UMESC**.

Upon receipt of the 3D Vision 120 Hz monitor (Samsung 2233RZ) [51], the team loaded the drivers for the display and USB IR emitter, and were immediately impressed with the monitor's resolution (1680 × 1050 pixels) and the stereo effect of the 3-D demos included with it. They then waited for the driver that would support the full-colour (non-anaglyph), quad-buffering required for their stereo viewing GIS software. At last, and after much pleading from the online community, NVIDIA released a driver on 16 June 2009 supporting quad-buffered stereo on both XP and Vista (version 186.18). The simultaneously released 3D Vision stereo driver, however, was a Vista-only release. A Quadro FX 3700 [49] replaced the GeForce and the newly released drivers were installed. The emitter that controls the shutter glasses was immediately detected, but viewing any stereo applications caused the computer to lock up. Fortunately, this issue was resolved by moving the Quadro to the second PCI-E (Peripheral Component Interconnect Express) port, indicating the fault was with the computer and not the video card. Once switched, the stereo game demo movie, included in the 3D Vision driver installation, looked fantastic and the computer was stable. A Windows Vista-friendly demonstration version of Stereo Analyst for ArcGIS (SAfA [44]) was installed and the stereo SAfA demo data looked just as good. The stereo effect was immediate and flicker-free. Panning through the imagery was initially quite slow but these issues were resolved by adjusting various settings within the NVIDIA Control Panel. Installing the XP display driver did not enable the emitter, so the system defaulted to anaglyph. The team discovered the installation executable could be unzipped to reveal the files contained within the stereo driver executable. They were able to manually install the stereo driver under Windows XP by right-clicking the nvstereo.inf and nvstusb.inf and selecting 'Install' from the right-click menu. In addition, they registered the OGLStReg.reg and NvStDef.reg registry files by right-clicking each and selecting 'Merge'. Upon completion of these steps, the stereo emitter and shutter glasses worked on the XP installation of SAfA as well (Figure 8).

Since mid-June 2009, several more versions of the display and stereo drivers have been released and documentation for enabling quad-buffering is improving. On 24 September 2009, the first 3D Vision driver specifically for Quadro video cards was released. The USGS BRD UMESC team has discovered the optimum settings for 3D Vision through trial-and-error and these settings have resolved most hardware and software issues encountered earlier. The few that remain, primarily the computer occasionally freezing when exiting OpenGL (the language that enables quad-buffering), may have sources apart from 3D Vision. The team feels confident that these drivers, and the documents detailing their installation and configuration, will continue to improve and the future for on-screen digitizing of stereo aerial photography is bright.

## Conclusion

There is no doubt that stereoscopic ("true 3D") technologies have and will continue having many useful serious applications in a number of fields, including GIS [52,53]. In this paper, we have briefly reviewed a number of stereoscopic 3-D hardware and software solutions for creating and displaying online maps and virtual globes in "true 3D", with costs ranging from almost free (anaglyphs) to multi-thousand pounds sterling. The limited colour fidelity of red/cyan anaglyph images can be improved using the recently introduced TrioScopics green/magenta (right eye is magenta) stereo encoding technique [54]. Glasses-free solutions, including options for mobile device displays, and stereoscopic 3-D virtual globes with more natural multitouch navigation are already available today, but in the future we may well be able to also feel what we see by using stereoscopic 3-D displays with tactile feedback [55-57], and we might even gain some "bionic eyesight" by wearing special augmented reality contact lenses [58]!

## Competing interests

The authors declare that they have no competing interests.

## Disclaimer of non-endorsement

Reference herein to any specific commercial products, processes or services by trade name, trademark, manufacturer or otherwise does not necessarily constitute or imply its endorsement, recommendation or favouring by the United States Government or its employees. Views and opinions of authors expressed herein do not necessarily state or reflect those of the United States Government, and shall not be used for advertising or product endorsement purposes.

## Authors' contributions

MNKB conceived and wrote the manuscript, and created all figures, except Figures 7 and 8, which were contributed by LRR. LRR provided material on USGS BRD UMESC's experience in setting up a low-cost, full-colour stereoscopic 3-D system. Both authors read and approved the final manuscript. Commercial products and company/brand names mentioned in this paper are trademarks and/or registered trademarks of their respective owners.
